# Repeatability, reliability, and stability of eye movement measurements in Parkinson’s disease, cerebellar ataxia, and healthy adults

**DOI:** 10.3389/fneur.2025.1556314

**Published:** 2025-04-28

**Authors:** Solveig E. J. Dalbro, Ahmed Elsais, Siri Lynne Rydning, Mathias Toft, Emilia Kerty, Stig E. Larsen

**Affiliations:** ^1^Department of Neurology, Oslo University Hospital, Oslo, Norway; ^2^Institute of Clinical Medicine, University of Oslo, Oslo, Norway; ^3^Meddoc Research AS, Skjetten, Norway

**Keywords:** eye movement, saccades, smooth pursuit, cerebellar ataxia, Parkinson’s disease

## Abstract

**Introduction:**

Eye movements have been proposed as biomarkers to track disease progression and treatment effects in neurological diseases. Before such variables are used in the clinic or in drug trials, properties such as measurement error must be documented. In this study, we assessed repeatability, reliability, and stability of fixation, smooth pursuit, and saccade measurements in patients with Parkinson’s disease, cerebellar ataxia, and healthy adults.

**Methods:**

Fixation, smooth pursuit, and saccade metrics were measured in 16 patients with Parkinson’s disease, 16 patients with ataxia, and 25 healthy adults with an eye tracker (BulbiCam). The same operator repeated the measurements six times over 2 days in the patient group and two times the same day in the healthy adults. Reliability, repeatability, and stability were assessed with the intraclass correlation coefficient (ICC), Bland–Altman plots with the Agreement Index, and the Stability Index, respectively.

**Results:**

Mean pupil size in the fixation test and latency, accuracy and peak velocity in the pro-saccade test were found reliable, repeatable, and stable. Mean and max fixation in the fixation test were found reliable and stable. Smooth pursuit measurements were found repeatable within patients and stable, but not reliable.

**Conclusion:**

The saccade and pupil variables may be used both on a population level and for individual patient follow-up. Mean and max fixation duration may be used on the population level but used in the clinical evaluation on individual patients they need to be repeated.

## Introduction

Diseases affecting the nervous system are the leading cause of global disability, and the research effort to ease this burden is formidable (1). Finding effective treatment also involves the search for biomarkers. In Parkinson’s disease (PD), there are promising imaging and fluid biomarkers for early diagnosis and risk stratification (2). For cerebellar ataxias, the continual identification of novel genetic causes enhances diagnostic accuracy. However, there is a lack of biomarkers to track disease progression objectively in many neurological diseases.

Eye movement measurements are candidate biomarkers in neurological diseases as they give valuable information about the healthy and pathological brain. Eye movements can be objectively measured and quantified, and oculomotor abnormalities are well described in diseases such as Huntington’s disease (3), Alzheimer’s dementia (4), multiple sclerosis (5), stroke (6), PD (7–9), and cerebellar ataxia (10).

When searching for biomarkers and clinical variables that can track disease progression or monitor effect of interventions, the degree of measurement error is crucial. Repeatability and reliability studies provide information about this variable and strengthens the validity of the results. Repeatability is the degree to which repeated scores or ratings are identical, or the agreement, within subjects (11). Reliability is a term often used in measurements with clinical scales and questionnaires. It extends beyond repeatability within subjects to encompass repeatability both between and within subjects, but mainly between subjects. A reliable variable must be both repeatable between and within subjects. Reliability of a measurement depends on the variation between subjects and thus relates to the population of which the subjects can be considered a random sample. The intraclass correlation coefficient (ICC) is mostly used to express the reliability of a measurement on population level. The Bland–Altman plots and calculation of agreement limits are recommended for evaluating repeatability within subjects. Stability is a term used for repeatability of measurements over several time points and can reveal practice effects that may be relevant in longitudinal studies or clinical trials. A reliable and repeatable biomarker can be used both on a population level and for follow up of an individual subject. If the biomarker is reliable and stable in terms of high ICC, but not repeatable within patient, it can still be used on a population level but would need to be repeated for use on individual subjects. A within-subjects repeatable and stable but not reliable biomarker is not applicable in clinical studies on a population level but can be used in the follow-up of individual subjects.

Studies on reliability of fixation, smooth pursuit, and saccadic eye movements have been done in healthy individuals (12–16) with different eye-tracking devices. Except for a study of presymptomatic Huntington’s disease gene carriers (17), to our knowledge, there exist few studies on reliability of eye movement measurements in neurological patients. Reliability and repeatability of a measurement technique are not fixed, but a result of interactions between the equipment, the subjects measured, and the context of assessment. As neurological patients frequently have difficulties with fixating and limitations in their eye movements, calibration can be challenging. It is therefore important to know the reliability of these eye movements not only in the healthy population but also in the relevant patient population. We chose to examine patients with PD and ataxia as they have well described eye movement pathologies (18) and there is a lack of objective methods to monitor disease progression. We also examined healthy adults of the same age to evaluate whether the reliability and repeatability were disease specific and for comparison with previous studies in healthy adults.

To assess reliability and repeatability of fixation, smooth pursuit, and saccade measurements in these participants, we use ICC and Bland–Altman analysis, and we propose a novel Stability Index for repeated measurements.

## Methods

*The study population* consists of patients previously diagnosed with PD or hereditary or sporadic cerebellar ataxia of both genders, and healthy adults, passed the age of 18 years, without any eye disease and other known serious diseases. For complete inclusion and exclusion criteria, see Supplementary material.

*The study subjects* were recruited from the outpatient clinic of the Department of Neurology, Oslo University Hospital. All participants gave written informed consent. The study was approved by the institutions data protection officer of Oslo University Hospital and considered by the Regional Ethics committee to be exempt (REK Nord, application number 401897). The study followed the tenets of the Helsinki Declaration with ClinicalTrial.gov number NCT 05449041 and EudraCT number 2021-006250-31.

The PD subjects consisted of seven women and nine men with the mean age of 65 years (range: 45–80) and disease duration from diagnosis of 7 years (range: 0.5–23), while the cerebellar ataxia subjects consisted of eight women and eight men with the mean age of 56 years (range: 34–75) and mean disease duration of 10 years (range: 0.5–33). The PD patients and cerebellar ataxia subjects were analyzed as one group. The healthy adult group consisted of 12 women and 13 men with a mean age of 61 years (range: 33–80).

On clinical examination five of the ataxia patients had nystagmus, 12 saccadic smooth pursuit, and three noted as having hypometric saccades. Among the PD patients, seven were categorized as having saccadic smooth pursuit and six had hypometric saccades on clinical examination. All the healthy adults underwent normal clinical neuroophthalmological examinations.

### Study design

The study was performed as a controlled, but non-randomized stratified parallel group trial with six repeated measurements in the two patient groups and two measurements in the healthy group. The clinical diagnosis was used as a stratification factor.

### Equipment

The BulbiCam produced by BulbiTech (Trondheim, Norway) was used for eye movement recordings. The apparatus uses dark pupil/bright pupil and corneal reflex technique video-oculography with a frequency of 400 frames per second to produce gaze direction data. It contains two screens and one infrared eye-tracking camera. BulbiCam can show stimuli to one or both eyes and track one or both eyes depending on the test chosen. Further details about the apparatus and software are given in the Supplementary material. BulbiHub software versions 221,031 and 221,216 were used in the study.

### Clinical procedure

Participants were placed in a comfortable chair with backrest and armrest. The BulbiCam was suspended from the ceiling with a wire and the participants attached to the camera with a headband (for a detailed setup, see the Supplementary material). BulbiCam registrations were done three times a day on two consecutive days for the patients, and two registrations on 1 day for the healthy participants. Each registration took approximately 10 min and was repeated after a 50-min break. Calibration was done automatically by software of the machine with an 8-point saccade test for the fixation and smooth pursuit test and the saccade and prosaccades test has in-test calibration (more details in the Supplementary material). All registrations were done by the same operator. Participants were given standardized instructions in Norwegian before each task (Supplementary material). The BulbiCam tasks used in this study are as follows:

#### Fixation

A central green cross in a dot target recommended by Thaler et al. (19) was illuminated for 11 s and repeated four times with a 4-s break in between. BulbiHub software denotes any horizontal eye movement above a threshold of 30 degrees per second as a saccade. The time between these saccadic intrusions are given as mean fixation duration and max fixation duration in milliseconds. The mean pupil size during the task with dark grey screens is given in millimeters (mm). As the BulbiCam is attached to the participant, no light from the room affects the pupils during the task.

#### Prosaccades

A pro-saccadic step task with 20 trials designed according to the standardized anti-saccade protocol (20). A green cross in dot target moves 10 degrees horizontal from the center with randomized direction and duration of the foreperiod. The variables obtained from the prosaccade test were latency (ms), accuracy (%), and peak velocity (degrees/s).

#### Smooth pursuit

A green dot moves sinusoidally 8 degrees left and right of the center, first at a frequency of 0.2 Hz, and then at 0.5 Hz and then follows a sequence when the target moves one half cycle at 0.25 Hz, one half cycle at 0.33 Hz, two half cycles at 0.5 Hz, and then five half cycles at 1 Hz. The variables obtained from the smooth pursuit test were first gain (0.2 Hz), second gain (0.5 Hz), and third gain from the right eye given in percent.

#### Saccade

A prosaccadic step task that consists of five trials of horizontal 20 degrees, five trials of horizontal 7.5 degrees saccades and 5 trials of vertical 9 degrees prosaccades. Each of the three variables, latency, accuracy, and peak velocity, was obtained for horizontal 20-degree, horizontal 7.5-degree, and vertical 9-degree saccades.

#### Eye movement analysis

The analysis was done using BulbiHub software with automatic blink removal and interpolation of missing glints and saccade detection. For the fixation task filter settings were set to moving average 1 (smoothing averaging one frame before and after the present frame) and velocity calculation using the smoothed gaze position compared with three frames before (N-3) and velocity threshold 30 degrees/s in BulbiHub software before export. Information on frequency filter in the software is not available from the manufacturer. For the smooth pursuit task, the results from the software were analyzed for the right eye. Software assigns 9 points for the first stimulus frequency, 5 points for the second stimulus, and 7 points for the third stimulus frequency and trough cross-correlation analysis calculates time-shift between phase of visual stimulus and phase of the eye movement, showing values for the gain in percent. With the step saccade task, we did not expect express saccades and therefore discharged trials with latencies <100 ms as technical errors or anticipatory saccades. The velocity threshold for saccade detection was 30 degrees/s. Trials with velocities above 700 degrees/s were regarded as technical errors and removed. The amplitude was assessed with amplitude accuracy, the measured saccade amplitude in relation to the target amplitude in percent. Trials with amplitude accuracy less than 10% were considered peristimulus fixation instability, instead of voluntary saccades, and therefore removed. The BulbiHub denotes trials with in-test calibration errors as amplitude gain of 120% instead of Na, and these trials were also removed.

### Statistical analysis

#### Repeatability and Reliability

A reliable variable on a population level needs to be repeatable both between and within participants. A reliable variable on an individual participant level needs to be repeatable and stable within participants. The performed reliability and repeatability analysis in this study includes both the intraclass correlation coefficient (3,1) (ICC) (21) and the Bland–Altman model (22, 23). The ICC was calculated with the two-way mixed-effect absolute agreement model, mainly focusing on repeatability between patients (24). The Bland–Altman model was used to express the repeatability or agreement within participants. This model is expressed graphically by the Bland–Altman plot, given as the mean difference between two measurements of the same object with a 95% prediction interval calculated as mean difference ± 2 times the standard deviation of the difference between the two measurements (SD_diff_). These intervals are referred to as Agreement limits. In addition, an Agreement Index (AI) (25) defined as 1 minus the ratio between the half width between the agreement limits and the measurement mean level is used. AI = 1–2*SD_diff_/Mean of the measurements. We used the following categorization of both AI and ICC (24):

- < 0.50—poor.
- [0.50, 0.75 > —moderate.
- [0.75, 0.90 > —good.
- [0.90, 1.00]—excellent.

#### Stability

Let SD_b_ and SD_w_ denote the standard deviation between and within participants, respectively. The ratio SD_w_/SD_b_ is considered a good indicator for stability. A low ratio indicates good stability and must be below 1 to claim stability. To obtain a stability index which increases with increased stability, we introduce the stability index as SI=1−SD_w_/SD_b_. In contrast to other classification indices where the classification limits are set without scientific justification, such as for ICC and AI, here it is desired to base the limits on probabilities. Let n denote the number of repeated observations within patients and m the number of patients. The ratio between two chi-squared distributions with (n-1) and (m-1) degrees of freedom (df) multiplied with the inverse df-ratio is Fisher-distributed with (n-1) and (m-1) df (26). Consequently, if you calculate the ratio between the sum of squares within patients ( ${S_{w}}^{2}$ ) and the sum of squares between patients ( ${S_{b}}^{2}$ ) and multiply this ratio by ((m–1)/(n–1)), the result follows an F-distribution with (n–1) and (m–1) degrees of freedom.

$\begin{array}{l} F=\left[{S_{w}}^{2}/{S_{b}}^{2}\;\right]\times \left[\left(m-1\right)/\left(n-1\right)\right]=F\;\left[{S_{w}}^{2}/\left(n-1\right)\right]/\\ \;\left[{S_{b}}^{2}/\left(m-1\right)\right]=F\;{\left[\;{SD}_{w}/{SD}_{b}\right]}^{2}\; \end{array}$ is Fisher-distributed with (n–1) and (m–1) df.

$\begin{array}{l} P\;\left\{\left[{\left({SD}_{w}/{SD}_{b}\right)}^{2}\right]\leq f_{1-\alpha }\right\}=1-\alpha \;\mathrm{gives that}\;\\ P\;\left[\left({SD}_{w}/{SD}_{b}\right)\leq \sqrt{f_{1-\alpha }}\;\right]=1-\alpha \; \end{array}$ and P (SI ≤ $\sqrt{f_{\alpha }})$ = α. Different choice of α gives the classification limits of the stability index SI.

In this study, we have *n* = 6 repeated observations and m = 32 patients which gives [SD_w_/SD_b_]^2^ F-distributed with 5 and 31 df for each patient. This gives the possibility to calculate and classify the stability for each patient (SI_i_; i = 1 to 32) on a given variable. SI_i_ may vary between patients, and the mean of the SIs may be given with confidence intervals for the total material. The *α*-values 0.05, 0.10, 0.20, and 0.40 provide the following classification limits:

1. Excellent stability (α=0.05) gives SI ≥ $1-\sqrt{f0.95}$ = $1-\sqrt{0.223}\;SI\geq$ 0.53
2. Very good stability (α=0.10) gives $1-\sqrt{f0.90}\leq S$ I< $1-\sqrt{f0.95}$ SI ≥ 0.44
3. Good stability (α=0.20) gives $1-\sqrt{f0.80}\leq S$ I < $1-\sqrt{f0.90}$ SI ≥ 0.34
4. Acceptable stability (α=0.40) gives $1-\sqrt{f0.60}\leq S$ I 1 – < $\sqrt{f0.80}$ SI ≥ 0.14
5. Poor stability (α>0.40) gives SI < $\sqrt{f0.60}$ SI < 0.14

Statistical analyses were performed in SAS version 9.4.

## Results

### Fixation

**Figure 1:**
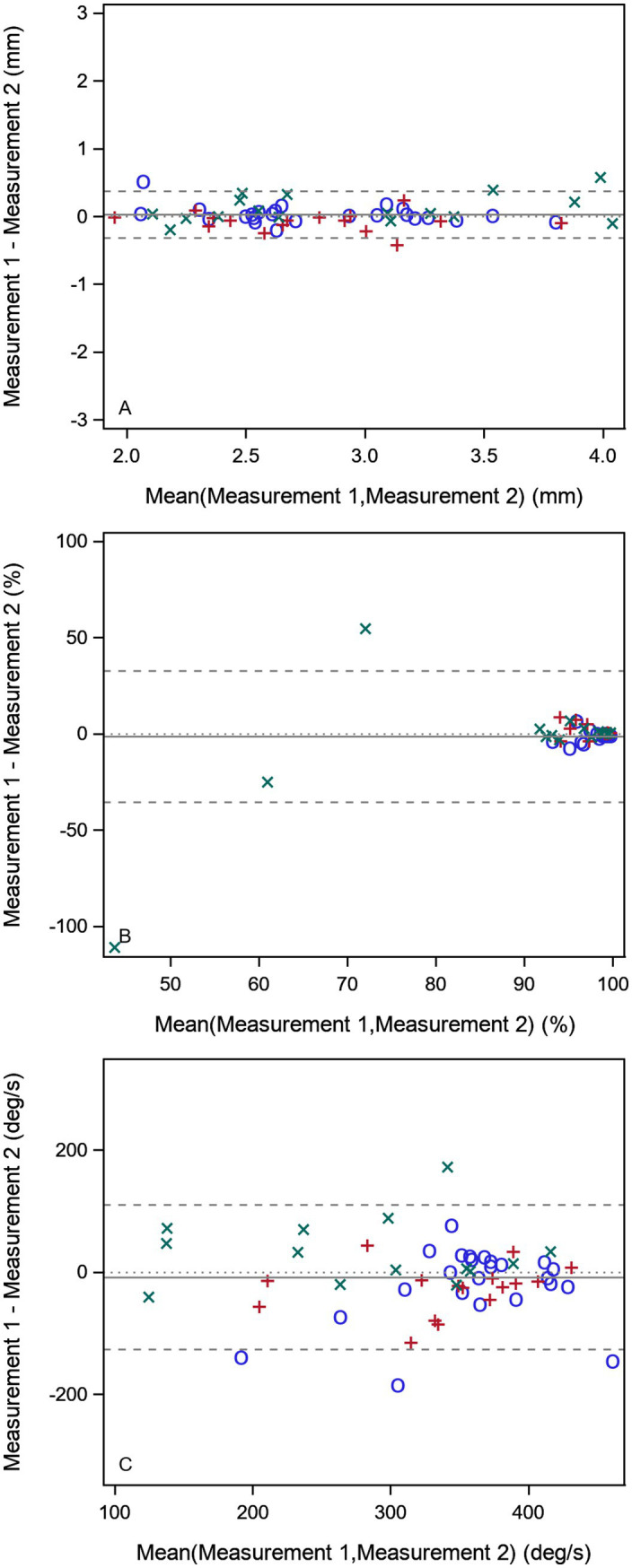
Bland–Altman plot of some selected variables from the fixation, smooth ursuit, and prosaccade test in the merged patient (*n* = 32) and healthy control (*n* = 25) material. **(A)** Mean pupil diameter in millimeters from the fixation task. **(B)** Smooth pursuit gain of first (0.2 Hz) stimuli. **(C)** Saccade peak velocity in degrees per second in the prosaccade task. The full horizontal line shows the mean difference between the two measurements, and the dotted horizontal lines indicate the agreement limits. The blue circles (○) shows healthy controls, the red plus (+) Parkinson patients, and the green cross (X) ataxia patients.

**Figure 2:**
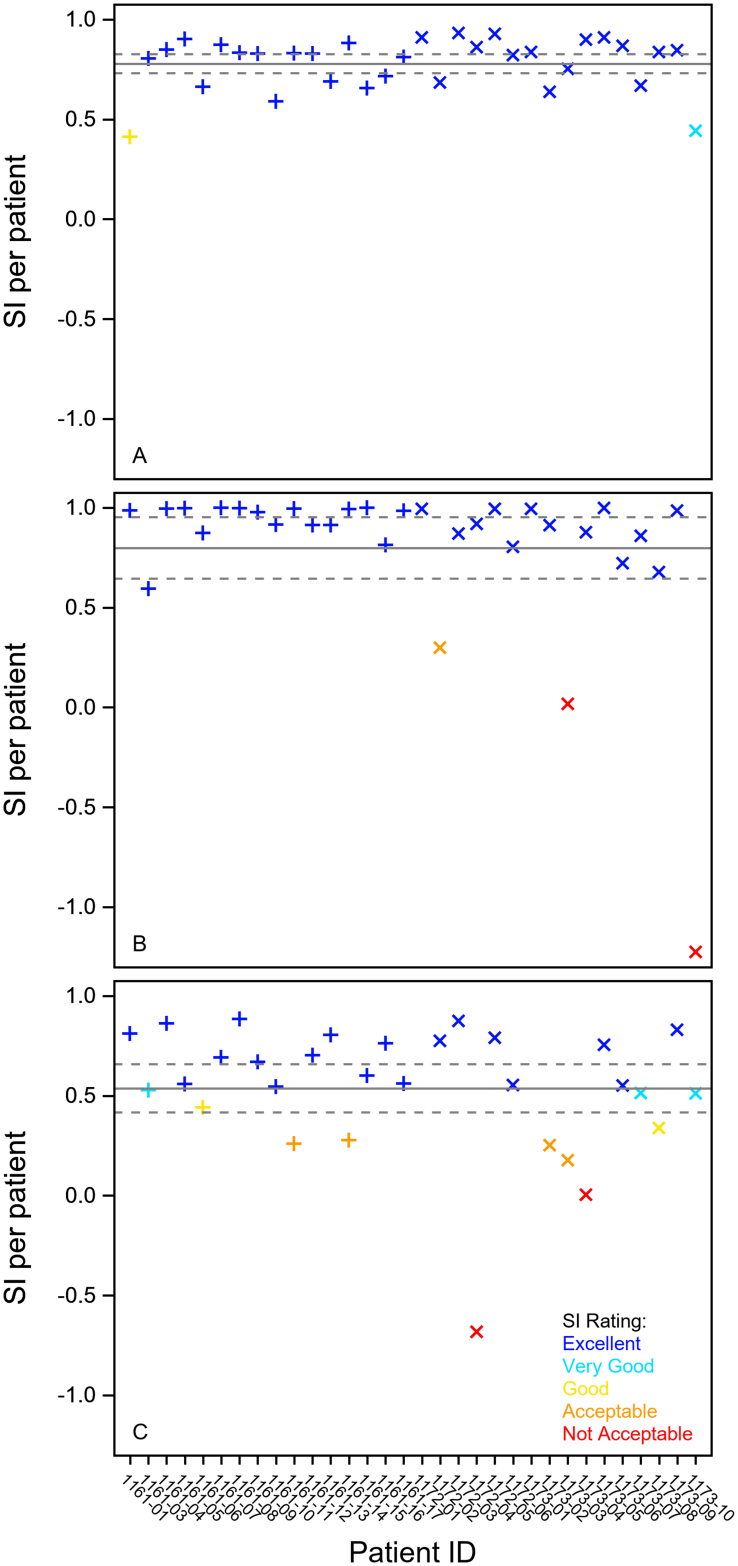
Stability plot of individual results for some selected variables from the fixation, smooth pursuit, and prosaccade test in 16 Parkinson patients (+) and 16 ataxia patients (X). **(A)** Mean pupil diameter in millimeters from the fixation task. **(B)** Smooth pursuit gain in percent (%) of first (0.2 Hz) stimuli. **(C)** Saccade peak velocity in degrees per second in the Prosaccade task. The full line shows the mean stability index, and the dotted lines indicate the 95% confidence interval. The individual stability index is given on the x-axis and the classification in different colors (blue—excellent, cyan—very good, yellow—good, orange—acceptable, red—not acceptable). SI Stability Index.

The pupil size variable in the fixation task was reliable, repeatable, and stable (Tables 1, 2; Figures 1A, 2A). Mean and max fixation duration were reliable for both patients and healthy controls. However, these fixation duration variables were not found repeatable but stable in both groups (Tables 1, 2).

**Table 1:** Reliability and repeatability of the BulbiCam tests.

Test	Variable	Patients (*n* = 32)				Healthy controls (*n* = 25)			
		First	Second	ICC	AI	First	Second	ICC	AI
Fixation	Mean fixations (ms)	1,311	1,378	0.63	−0.50	1,431	1,690	0.78	−018
	Max fixations (ms)	5,476	5,074	0.71	0.15	5,955	6,088	0.85	0.47
	Mean pupil (mm)	2.9	2.9	0.94	0.86	2.83	2.78	0.96	0.91
Smooth pursuit	First gain (%)	92.7	94.8	0.07	0.51	98.3	98.7	0.32	0.95
	Second gain (%)	91.2	91.7	0.18	0.80	93.1	95.7	0.31	0.86
	Third gain (%)	82.6	81.9	0.30	0.78	89.1	88.1	0.20	0.75
Pro saccade	Latency (ms)	283.7	292.0	0.50	0,57	277.0	285.7	0.45	0.66
	Accuracy (%)	83.8	87.5	0.75	0,72	91.1	94.3	0.28	0.73
	Peak velocity (deg/s)	312.8	313.6	0.80	0,64	352.2	370.4	0.54	0.66
Saccade latency (ms)	Horizontal 20 deg.	301.6	310.5	0.41	0.53	277.0	278.5	0.42	0.70
	Horizontal 7.5 deg	236.3	230.3	0.76	0.73	228.6	219.8	0.79	0.81
	Vertical	251.2	270.3	0.46	0.59	260.5	261.2	0.89	0.82
Saccade accuracy (%)	Horizontal 20 deg.	76.9	82.4	0.00	0.58	82.8	85.8	0.23	0.67
	Horizontal 7.5 deg.	81.7	90.3	0.36	0.56	85.9	84.6	0.36	0.66
	Vertical	89.5	84.2	0.10	0.44	97.8	97.8	0.15	0.65
Saccade peak velocity (deg/s)	Horizontal 20 deg.	334.8	347.1	0.71	0.55	410.7	406.5	0.61	0.63
	Horizontal 7.5 deg.	293.6	320.7	0.39	0.55	344.6	336.6	0.28	0.67
	Vertical	318.8	295.8	0.59	0.52	377.8	372.5	0.61	0.71

Reliability related to population is expressed by the intraclass correlation (ICC) and the repeatability within patients by the Agreement Index (AI). The results are expressed by mean value. Ms, milliseconds; mm, millimeters; Hz, Hertz; %, percent; deg, degrees; deg/s, degrees per second.

**Table 2:** Stability of the BulbiCam tests.

Test	Variable	M1	M2	M3	M4	M5	M6	ICC	SI (95%CI)	Classification
Fixation	Mean fixations (ms)	1,311	1,378	1,486	1,612	1,397	1708	0.56	0.33 (0.08–0.57)	Good
	Max fixations (ms)	5,476	5,074	5,549	5,673	5,357	5,286	0.62	0.48 (0.37–0.59)	Very good
	Mean pupil (mm)	2.9	2.9	2.9	2.8	2.8	2.8	0.94	0.78 (0.73–0.83)	Excellent
Smooth pursuit	First gain (%)	92.7	94.8	96.5	96.9	97.4	94.8	0.24	0.80 (0.64–0.95)	Excellent
	Second gain (%)	91.2	91.7	89.6	89.6	91.7	91.4	0.18	0.27 (−0.05–0.58)	Acceptable
	Third gain (%)	82.6	81.9	82.2	79.9	79.9	80.1	0.31	−0.04 (−0.34–0.27)	Not acceptable
Prosaccade	Latency (ms)	283.7	292.0	292.5	295.2	290.7	292.5	0.56	0.52 (0.36–0.68)	Very good
	Accuracy (%)	83.8	87.5	89.4	86.7	84.3	86.0	0.61	0.53 (0.40–0.67)	Excellent
	Peak velocity (deg/s)	312.8	313.6	337.8	320.3	318.6	320.2	0.69	0.54 (0.42–0.66)	Excellent
Saccade latency (ms)	Horizontal 20 deg	301.6	310.5	287.0	294.3	294.1	329.3	0.40	0.31 (0.05–0.57)	Acceptable
	Horizontal 7.5 deg.	236.3	230.3	240.6	238.7	229.9	230.9	0.50	0.44 (0.29–0.59)	Very good
	Vertical	251.2	270.3	252.9	252.7	256.5	265.9	0.47	0.18 (−0.11–0.47)	Acceptable
Saccade accuracy (%)	Horizontal 20 deg.	76.9	82.4	79.5	79.2	78.5	80.2	0.23	0.31(0.15–0.47)	Acceptable
	Horizontal 7.5 deg.	81.7	90.3	84.1	82.8	81.6	77.4	0.35	0.37(0.20–0.54)	Good
	Vertical	89.5	84.2	83.5	81.1	82.9	89.7	0.18	0.08(−0.08–0.24)	Not acceptable
Saccade peak velocity	Horizontal 20 deg.	334.8	347.1	343.8	357.5	329.7	338.1	0.73	0.51(0.42–0.61)	Very good
	Horizontal 7.5 deg.	293.6	320.7	308.4	296.0	288.4	286.6	0.51	0.34(0.18–0.49)	Good
	Vertical	318.8	295.8	300.8	296.0	289.5	315.1	0.53	0.28 (0.17–0.39)	Acceptable

The six measurements are denoted as M1 to M6 and expressed by mean values. Stability is expressed by the Stability Index (SI) with 95% Confidence Intervals (CI). The classification is based on SI. Ms, milliseconds; mm, millimetre; Hz, Hertz; deg, degrees; deg/s, degrees per second.

### Smooth pursuit

None of the three smooth pursuit variables were found reliable neither in the patients nor the controls (Table 1). However, all these variables were repeatable (Table 1, Figure 1B) and 0.2 Hz and 0.5 Hz gain were stable (Table 2; Figure 2B).

### Prosaccades

Latency, accuracy, and peak velocity in the prosaccades task were all found reliable, repeatable, and stable in the patient group (Tables 1, 2; Figure 1B; 2C). In the healthy group, only the peak velocity was reliable, but all three variables were repeatable.

### Saccades

In the saccade task with vertical, short, and long horizontal saccades, the peak velocity of the short and vertical saccades as well as the latency of the short saccades were found reliable, repeatable, and stable (Tables 1, 2). Latency of the long saccades and the accuracy of both short and long horizontal saccades were found repeatable (Table 1) and stable (Table 2). Additional Bland–Altman plots and stability plots are found in the Supplementary material.

### Classification

Seven variables were classified as reliable, repeatable, and stable, seven as repeatable and stable, but not reliable (Table 3).

**Table 3:** Merged summary of reliability, repeatability, and stability.

Test	Variables	Parkinson and ataxia patients			Healthy controls		Merged classification
		ICC	AI	SI	ICC	AI	
Fixation	Mean fixations	+	−	+	+	−	Reliable + stable
	Max fixations	+	−	+	+	−	Reliable + stable
	Mean pupil	+	+	+	+	+	Reliable + repeatable + stable
Smooth pursuit	First gain	−	+	+	−	+	Repeatable + stable
	Second gain	−	+	+	−	+	Repeatable + stable
	Third gain	−	+	−	−	+	Repeatable
Pro Saccade	Latency	+	+	+	−	+	Reliable + repeatable + stable
	Accuracy	+	+	+	−	+	Reliable + repeatable + stable
	Peak velocity	+	+	+	+	+	Reliable + repeatable + stable
Saccade latency	Horizontal 20	−	+	+	−	+	Repeatable + stable
	Horizontal 7.5	+	+	+	+	+	Reliable + repeatable + stable
	Vertical	−	+	+	+	+	Repeatable + stable
Saccade accuracy	Horizontal 20	−	+	+	−	+	Repeatable + stable
	Horizontal 7.5	−	+	+	−	+	Repeatable + stable
	Vertical	−	−	−	−	+	Not acceptable
Saccade peak velocity	Horizontal 20	+	+	+	+	+	Reliable + repeatable + stable
	Horizontal 7.5	−	+	+	−	+	Repeatable + stable
	Vertical	+	+	+	+	+	Reliable + repeatable + stable

Significant values of reliability (ICC), repeatability (AI), and stability (SI) for each test variable are given with (+).

## Discussion

Eye movement measurements have been proposed as clinical biomarkers in neurological diseases. The validity of such biomarkers depends on the amount of measurement error and should be investigated in a relevant sample. In this study, we have assessed test–retest reliability, repeatability, and stability of fixation, smooth pursuit, and saccade measurements in a group of patients with suspected oculomotor abnormalities and healthy adults.

The fixation task variable pupil size was the most reliable, repeatable, and stable of the analyzed variables. The reliability and repeatability of a measurement is a product of interactions between the recording equipment, the subjects, and the measurement setting. The fixation task is undemanding to the participant and the illumination presented to the pupil from the apparatus and the task is constant, and we therefore expect the pupil to be of stable size during this short task. With recording equipment sampling at 400 frames per second, this variable offers a robust data set less susceptible to outliers. We interpret the good results in pupil measurements as low technical measurement error by the equipment and excellent reliability and stability of this variable means that it could be used in both cross-sectional trials and longitudinal trials.

The mean and max fixation duration were reliable on a group level assessed with ICC. However, the repeatability within patients was judged as poor by the Agreement Index, indicating greater variability within the individual than between individuals. The Bland–Altman plot shows that some participants have great variation between repeated measurements, and this is seen in both healthy individuals and patients. This means that fixation duration could be used to categorize or differentiate between groups in cross-sectional studies, but this test should be used with caution when following up the individual patient or in longitudinal trials where repeatability is crucial. The stability of all six measurements in the patients were however very good, indicating that increased recording time or number of cycles would likely improve the repeatability.

The smooth pursuit measurements showed poor reliability for all three stimuli speeds. This task design is prone to measurement error and susceptible to outliers because of few data points as it only includes nine, five, and seven data points (first, second, and third stimulus, respectively) to calculate the gain value. However, the repeatability was good and the stability excellent for the 0.2 Hz and acceptable for the 0.5 Hz gain. This means that for follow-up of the individual patient in the clinic or in longitudinal trials this task has the potential to detect changes from one visit to another, but the current task is not suitable for studies on a population level.

The saccade measurements in the prosaccade task showed good to excellent reliability and repeatability in the patient group. This test is applicable both on a population level and for the individual patient in clinical follow-up. In the healthy group, the latency and accuracy variables showed poor reliability. This discrepancy could be because subject variability can cause unreasonably low or high ICC values when measurement errors are fixed. Six of the ataxia patients, three of the PD patients, and none of the healthy adults had hypometric saccades on clinical examination. The higher ICC in patient saccade amplitude accuracy versus the healthy group is likely due to the lack of variability within the healthy participants and the great heterogeneity in the patient group. We note that limitation of software of saccade amplitude accuracy of 120% warrants caution when analyzing cohorts with hypermetric saccades, and preferably, raw data should be analyzed in this setting. The saccade task with short, long, and vertical saccades were overall repeatable and stable. However, the reliability varied with parameter and saccade length and direction, probably due to the low number of trials.

Comparison between studies is difficult, as differences in tasks, recording equipment, statistical approach, and participants influence the results and conclusions. Our prosaccade task was designed with stimulus size and presentation following the internationally standardized antisaccade protocol (20), but with fewer trials. This protocol was also used in a reliability study of saccade measurements in healthy adults by Plomecka et al. (12), which reported ICC values between 0.58 and 0.87 in the older group of participants, with lower ICCs in the younger participants. In our study, we observed higher ICCs in the patient group than the healthy group. This disparity might stem from the variability in the groups as we expect more saccade abnormalities and hence variability with older age and neurological disease (27). Ettinger et al. (15) also found that saccade and fixation measurements are reliable, but with different task designs. While the ICCs reported in both Plomecka et al. (12) and Ettinger et al. (15) are comparable, they are not identical to those we observed. The eye-tracking device differed across these studies: Plomecka et al. utilized the EyeLink 1000Plus (SR-Research, Ottawa, Canada), while Ettinger et al. employed an IRIS model 6,500 (Skalar Medical BV, Delft, Netherlands). Although BulbiCam has similar technical capabilities, there are clear differences in the technical setup (head-mounted/table mounted) as well as hardware and software configurations. As BulbiCam is equipped with preprogrammed tests and built-in analysis, assessing the variations in eye-movement analysis is challenging.

Standardized task protocols have been published like “The Internationally Standardized Antisaccade protocol” (20) and the DEMoNS protocol for saccade measurements (28). The Ataxia Global Initiative Working Group on Digital-Motor Biomarkers published in 2023 recommendations on task design for eye tracking studies in ataxia patients. Standardization of eye tracking protocols and reporting is crucial for exploiting the full potential of eye movements as a biomarker. In addition to these task protocols (10, 20, 28), Dunn et al. have published guidelines for reporting eye-tracking research across disciplines (29). We recommend implementing standardized task designs and transparent protocols in future studies. However, the trade-off between enough trials for reliability and repeatability on the one hand must be weighed against the feasibility in the relevant patient population in terms of cooperation and fatigue.

A strength of this study is the various statistical approaches to judge the measurement’s reliability, repeatability, and stability. Most reliability studies use ICC (12, 15), but other statistical methods (13–16) have also been used. As ICC is a measure of correlation, it can show excellent reliability even though the repeated measurements do not show repeatability. In longitudinal trials and in individual patient follow-up, a biomarker without repeatability is of little value. We have supplemented the ICC with a repeatability analysis both graphically with Bland–Altman plots and calculated an Agreement Index. ICC can also be used as a measure of stability. However, the limits of categorization into poor, moderate, good, or excellent are only arbitrary and not based on statistical probability. ICC does not give information about the stability of the measurements within an individual. We therefore suggested the Stability Index with categorization based on probabilities.

### Our study has some limitations

The sample size is moderate and could limit some of the analysis. The patient cohort is very heterogeneous as the ataxia group includes participants with different genetic and sporadic aetiologies. Furthermore, PD patients were in different stages of the disease and used different treatments that may alter eye movements. The reliability, repeatability, and stability of the studied parameters may be different in a more homogeneous sample, but our results give an implication of what parameters to study further. The study is also limited to a single operator, and future research would benefit from evaluating inter-rater reliability, particularly for application in multicenter trials. This study does face limitations due to the constraints of the eye-tracking system used. The calibration setup is non-customizable, and detailed operational information is not available, presenting challenges particularly for patients with nystagmus or fixation instability, as standard calibration may not account for these conditions effectively. Moreover, filter and analysis settings of software are not under full operator control, limiting comprehensive understanding and replication of findings with other devices. These limitations should be weighed against the benefits of using an eye-tracking system that is designed for ease of use in clinical settings.

## Conclusion

Eye movement measurements have repeatedly been promoted as promising biomarkers but lack the standardization and thorough validation to be utilized in randomized clinical trials and in the clinic. Our study has evaluated test–retest reliability and repeatability and has proposed a new method for evaluating stability with the Stability Index. We find that the saccade and pupil measurements in our study are reliable, repeatable, and stable in PD and ataxia patients. We recommend choosing oculomotor parameters appropriate to the study design with reliable parameters in cross-sectional studies and parameters with good repeatability and stability for longitudinal study designs and in the clinic for individual patient follow-up.

## Data Availability

The datasets presented in this article are not readily available due to legal restrictions, as they contain information that could compromise the privacy of research participants. Requests to access the datasets should be directed to soejac@ous-hf.no.
