# Supplementary material for: Repeatability, reliability, and stability of eye movement measurements in Parkinson’s disease, cerebellar ataxia, and healthy adults
Source: Front Neurol. 2025 Apr 28;16:1556314. doi: 10.3389/fneur.2025.1556314 (PMC12068062; doi:10.3389/fneur.2025.1556314)
Supplement: Supplementary file 1 [file Table_1.docx]

Supplementary Material

# Supplementary Data

**Methods**

*The study population:*

Inclusion and exclusion criteria:

*Inclusion criteria*

A Diagnosed with Parkinson disease OR hereditary or idiopathic sporadic cerebellar ataxia OR healthy adult.

B Passed the age of 18 years

C Without any other concomitant eye diseases

___________________________________________________________________________

*Exclusion criteria*

1. Other visual disturbances and blindness

2. Posterior Chamber Intraocular Lens (PCIOL)

3. Physical or psychiatric disease disturbing the measuring procedure

4. Paresis or paralysis of any oculomotor muscle

5. Not able to perform full eye movements

6. Visual acuity is less than 0.1 in any eye

7. Visible part of the eye is abnormal (haemorrhages/deformed pupils)

8. Pupils not able to respond normally to dilation or contraction

9. Participating in another clinical trial with pharmaceuticals

10. Not able to understand information

11. With known alcoholic and drug dependency

12. Not able to understand information

13. Not willing to give written consent to participate in the study

*Equipment***:**

The BulbiCam apparatus is built up by:

- two screens (LS055R1SX04 Sharp 5.5 2660x1600 @ 60 fps; 1920x1080 @60fps), two mirrors and behind the mirrors an infrared light and a camera. The camera produces alternating Dark pupil and Bright Pupil images and glint detection at a frequency of 400 FPS.


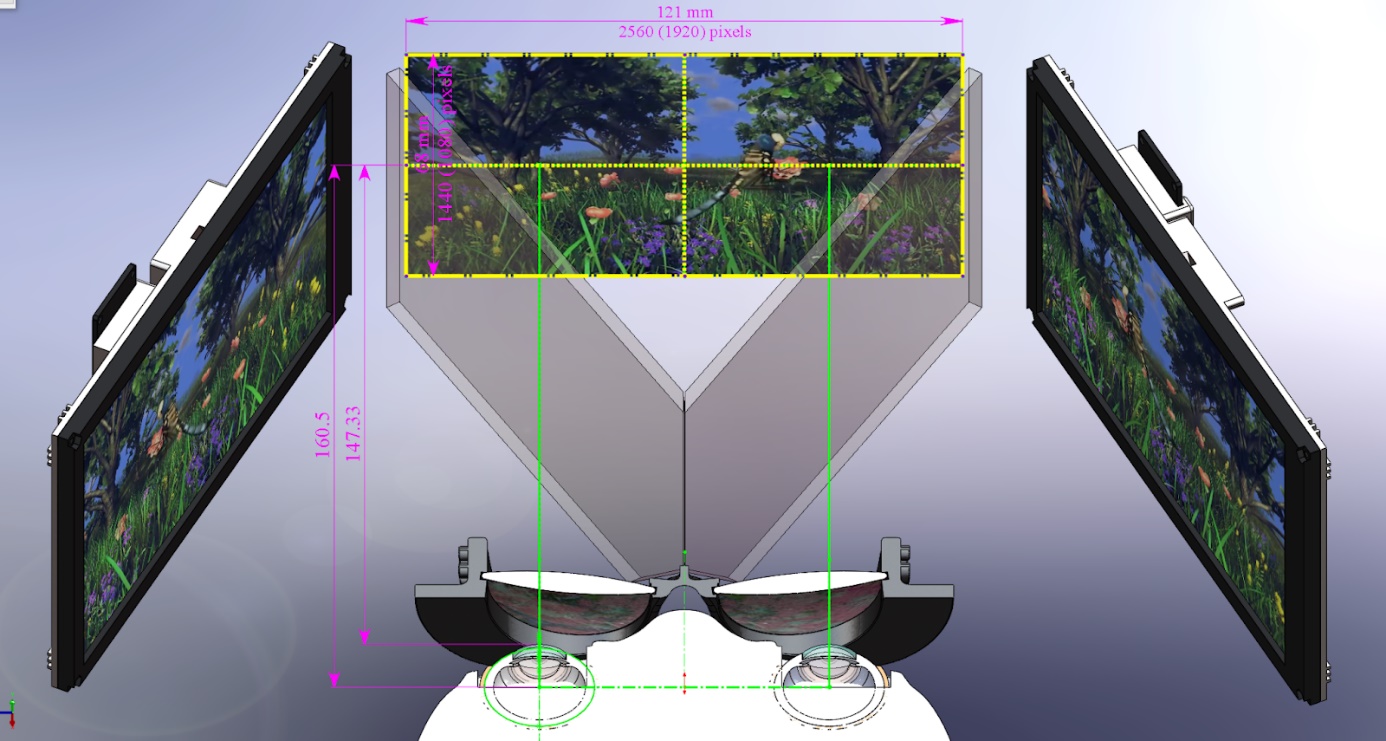


*Clinical procedure:*

The examinations were performed in a well-lit room in our out-patient clinic, but when the participant was attached to the camera only light from the monitors inside BulbiCam affected the pupils. Participants were placed in a comfortable chair with back and armrests. A magnetic mask was attached to the participants head with an elastic headband. The mask clicked into by magnetism the BulbiCam suspended from the ceiling. The head was unrestrained, but the participants were asked to sit still during the examinations.

Participants pupil distance and refraction was entered into the BulbiHub software. If the participant wore glasses, their correction for far vision + (+6) were used, for all other participants a +6 lens was put in the camera.

Calibration:

First the “functional screening”, the BulbiCam own calibration program was run. This includes both smooth pursuit eye movements and an 8-point saccade test with a green stimulus that produces a degree/glint pixel ratio used in the Fixation and Smooth Pursuit tests. The functional screening shows a green dot for recommended tests and an orange dot if the test has a risk to fail due to difficulties detecting the pupil or for the participant to follow the target. In the case of an orange or red dot, the functional screening was run up to three times to get a green dot. There was no feedback from the participant during the calibration. No other calibration validation was used in this study and no participant was excluded because of calibration difficulties at the functional screening. There were no recalibrations after the initial Functional Screening.

In the Saccades and Prosaccades test the software uses in test calibration with the assumption that the patients fixates at the stimulus at the end of each trial. Calibration errors in the Prosaccade test gives Amplitude Accuracy values of 120% and have therefore been removed in the analysis after eye movement recordings.

The initial functional screening was used for all the 6 examinations. The fixation, smooth pursuit and the prosaccade test uses data from the functional screening as test calibration. For the saccades test the system uses in test calibration where the system calibrates at the end of each stimulus assuming the participant is always looking at the stimulus at the end of the saccade. Further details on the calibration are not available in the manufacturer’s brochure.

The tasks were done in the following order each time: Smooth pursuit, saccades, fixation and prosaccades.

Patient instructions translated from Norwegian:

Functional screening: “First you see two green dots, then one. Look at them and follow as they move across the screen. Try not to blink.”

Smooth pursuit: “Follow the green dot.”

Saccades: “Follow the green dot.”

Fixation: “Focus on the green dot as accurately as you can. It will light up for 11 seconds at a time. There will then be a short pause where you can blink before the dot lights up again.”

Pro-saccade: “Look at the central dot; as soon as a new dot appears on the left or right, look at it as fast as you can.”

*The tasks:*

Fixation:

Green cross in dot of 0,5 degrees of visual angle (according to Thaler (1)) on a grey (128RBG) background shown to the left eye. Only left eye is recorded. The target was illuminated for 11 seconds with 4 seconds break, repeated 4 times giving 44 seconds of recordings.

Analysis: The first second is automatically removed from analysis by the software. The BulbiHub default settings are Average: 2, Glints:5 and Velocity threshold 15 degrees/s. This was changed to Average:1 (moving average) and Glints: 3 (the number of glints used in the calculation of velocities), and a velocity threshold of 30 degrees/s. Only horizontal eye movements are presented and analysed.

Smooth pursuit:

A green dot of about 0,63 degrees of visual angle on black background moving pendularly 8 degrees left and right of the centre, first at 0.2Hz (max speed 6.4 degrees/s), then 0.5 Hz (max speed 16 degrees/s), then with increasing speed from 0 to 22 degrees/s. Stimuli is presented to both eyes and both eyes are tracked.

Analysis: The software shows graphs of target versus eye movements for each eye. It also calculates gain values for each of the speeds. For this paper we analysed only right eye gain.

Pro Saccade:

The task is constructed in accordance with the protocol proposed by Antoniades et al (2) with a stimulus of 0,5 degrees of visual angle (green cross in dot) on a light grey background (128RGB). A foreperiod of 1-3,5sec (mean 1,5 seconds) in a non-ageing algorithm. Peripheral target duration 1 sec. Dominant eye tracked, default value right eye.

Analysis: The software does automatic saccade detection (based on velocity-threshold of 30deg/s) and provides measurement of latency, amplitude accuracy (in percentage) and peak velocity of the first saccade. We removed saccades with accuracy equal and above 120% as these often coexisted with missing data and accuracy less than 10% as these were assumed to be fixation instability rather than true voluntary prosaccades. We also removed saccades with latencies less than 100ms or peak velocity >700deg/s.

Saccades:

A green dot of 0,63 degrees of visual angle on black background moves first vertically 9 degrees 5 times, then horizontally 7,5 degrees 5 times, then 20 degrees 5 times.

Analysis: Saccade latency, amplitude accuracy and peak velocity is calculated by the software’s algorithm for each trial.

**References**

1. Thaler L, Schütz AC, Goodale MA, Gegenfurtner KR. What is the best fixation target? The effect of target shape on stability of fixational eye movements. Vision Res. 2013;76:31-42.

2. Antoniades C, Ettinger U, Gaymard B, Gilchrist I, Kristjánsson A, Kennard C, et al. An internationally standardised antisaccade protocol. Vision Res. 2013;84:1-5.

**Supplementary figures**

Supplementary figure 1


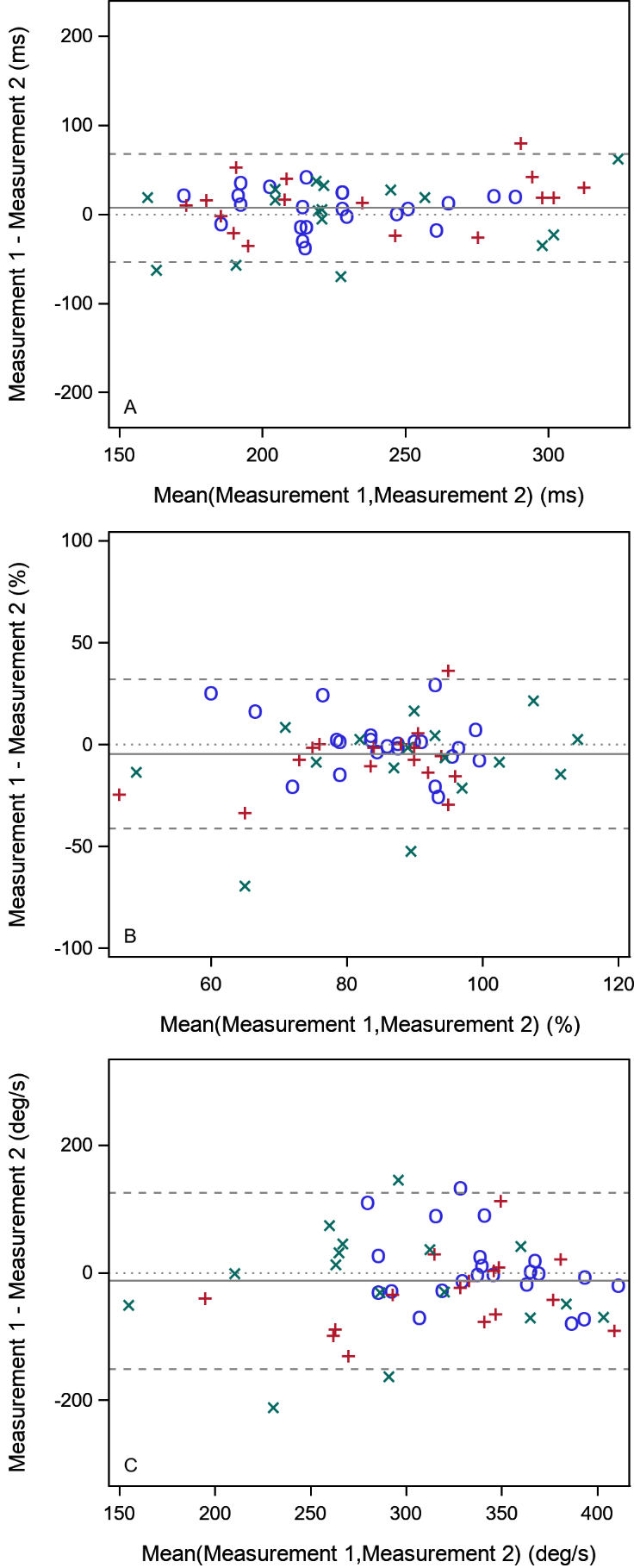


Supplementary figure 1. Bland-Altman plot of some selected variables from the Saccade-test in the merged patient (n=32) and healthy control (n=25) material. (A) Saccade latency in ms for horizontal 7.5 degrees saccade. (B) Saccade accuracy in percent for horizontal 7.5 degrees saccade. (C) Saccade peak velocity in degrees per second for the horizontal 7.5 degrees saccade. The full horizontal line shows the mean difference between the two measurements and the dotted horizontal lines indicate the agreement limits. The blue circles (○) shows healthy controls, the red plus (+) Parkinson patients and the green cross (X) ataxia patients.

Supplementary figure 2


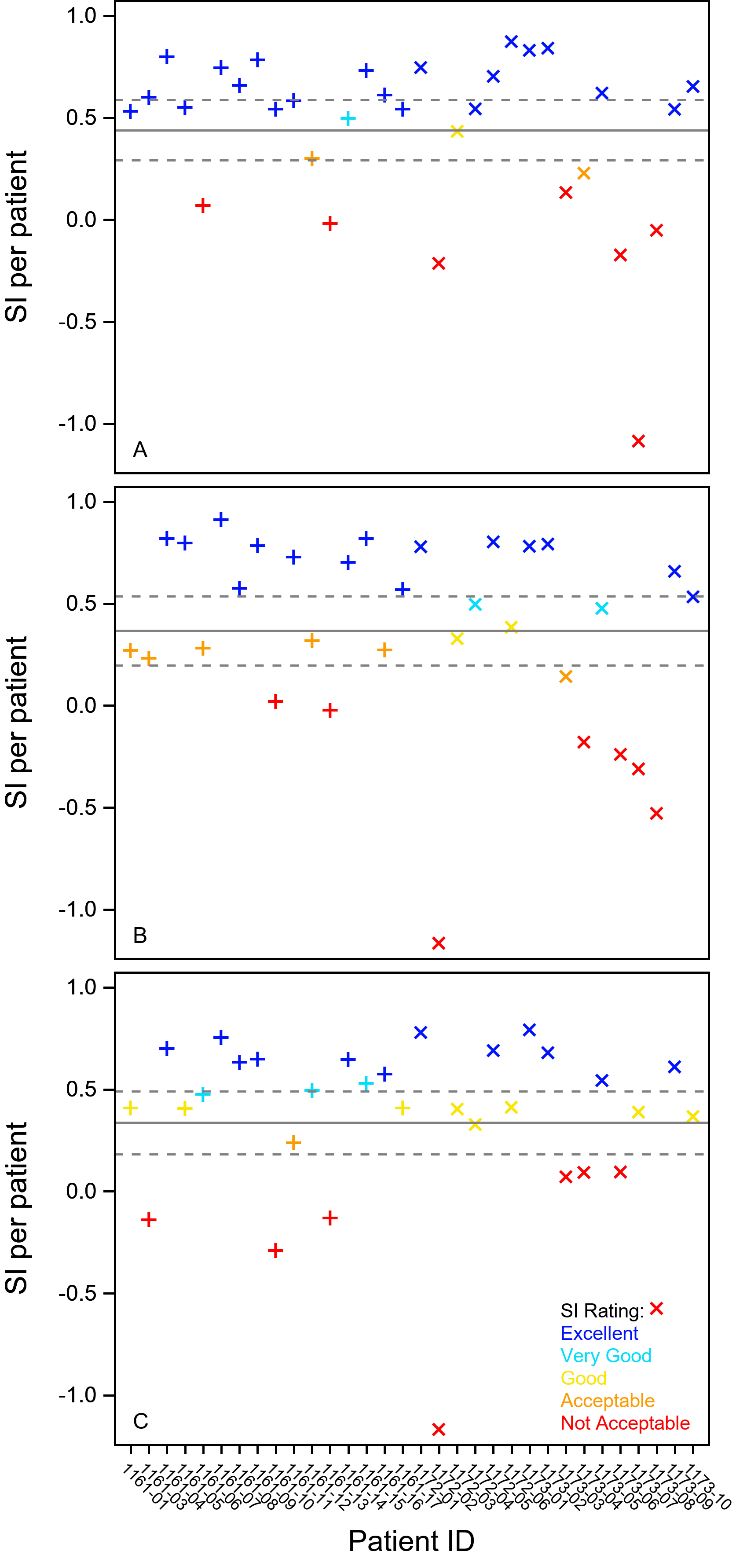


Supplementary figure 2. Stability plot of individual results for some selected variables from the Saccade test in 16 Parkinson patients (+) and 16 Ataxia patients (X). (A) Saccade latency in ms for horizontal 7.5 degrees saccade. (B) Saccade accuracy in percent for horizontal 7.5 degrees saccade. (C) Saccade peak velocity in degrees per second for the horizontal 7.5 degrees saccade. The full line shows the mean stability index, and the dotted lines indicate the 95% confidence interval. The individual stability index is given on the x-axis and the classification in different colours (Blue- excellent, cyan- very good, yellow- good, orange- acceptable, red- not acceptable). *SI stability Index.*
